# In Vitro Investigation of 3D Printed Hydrogel Scaffolds with Electrospun Tidemark Component for Modeling Osteochondral Interface

**DOI:** 10.3390/gels10110745

**Published:** 2024-11-15

**Authors:** Victoria Effiong Effanga, Dana Akilbekova, Fariza Mukasheva, Xiao Zhao, Dilhan M. Kalyon, Cevat Erisken

**Affiliations:** 1 Department of Chemical and Materials Engineering, School of Engineering and Digital Sciences, Nazarbayev University, 010000 Astana, Kazakhstan; victoria.effanga@nu.edu.kz (V.E.E.); dana.akilbekova@nu.edu.kz (D.A.); fariza.mukasheva@nu.edu.kz (F.M.); 2 Department of Chemical Engineering and Material Science, Stevens Institute of Technology, Hoboken, NJ 07030, USA; xzhao22@stevens.edu (X.Z.); dkalyon@stevens.edu (D.M.K.)

**Keywords:** osteochondral, 3D printing, tidemark, electrospinning, tissue engineering

## Abstract

Osteochondral (OC) tissue plays a crucial role due to its ability to connect bone and cartilage tissues. To address the complexity of structure and functionality at the bone–cartilage interface, relevant to the presence of the tidemark as a critical element at the bone–cartilage boundary, we fabricated graded scaffolds through sequential 3D printing. The scaffold’s bottom layer was based on a gelatin/oxidized alginate mixture enriched with hydroxyapatite (HAp) to create a rougher surface and larger pores to promote osteogenesis. In contrast, the upper layer was engineered to have smaller pores and aimed to promote cartilage tissue formation and mimic the physical properties of the cartilage. An electrospun ε-polycaprolactone (PCL) membrane with micrometer-range pores was incorporated between the layers to replicate the function of tidemark—a barrier to prevent vascularization of cartilage from subchondral bone tissue. In vitro cell studies confirmed the viability of the cells on the layers of the scaffolds and the ability of PCL mesh to prevent cellular migration. The fabricated scaffolds were thoroughly characterized, and their mechanical properties were compared to native OC tissue, demonstrating suitability for OC tissue engineering and graft modeling. The distance of gradient of mineral concentration was found to be 151 µm for grafts and the native OC interface.

## 1. Introduction 

Osteochondral (OC) tissue comprises both cartilage and the underlying subchondral bone and is critical in maintaining knee joint functionality. Damage to this tissue, whether due to trauma, aging, or degenerative diseases, can severely impair joint mobility and lead to chronic pain and disability, as in the case of osteoarthritis. Osteoarthritis is a progressive joint disorder characterized by the degradation of articular cartilage, which is very common among the elderly and affects over 250 million people worldwide [1]. However, OC tissue damage is almost irreversible due to the avascularity of articular cartilage, which severely limits its intrinsic healing capacity [2].

**Scheme 1:**
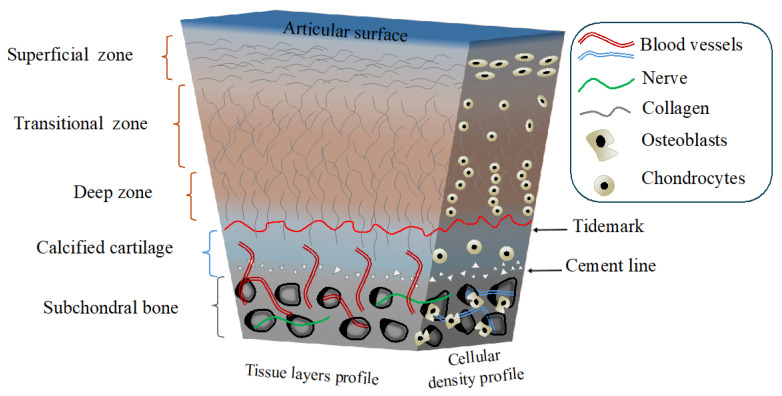
Graphical representation of osteochondral tissue, indicating three main regions: subchondral bone (SB)—gray, calcified cartilage (CC)—blue, and uncalcified cartilage (UC)—orange.

The osteochondral tissue is located at the distal ends of bones connected to joints. OC tissue contains three distinct yet gradually transitioning layers: the uncalcified cartilage (UC), calcified cartilage (CC), and subchondral bone (SB) [3,4]. The UC and CC are separated by a structure known as the tidemark, which is thought to be a remnant of the original growth plate [5]. Scheme 1 provides a representative illustration of the osteochondral tissue. Structurally, the tidemark is a 5–10 µm-thick semipermeable boundary that allows the passage of nutrients, waste products, and signaling molecules while helping to maintain the avascular nature of the UC with its unique functions of providing a smooth surface for joint movement and maintaining the connection to the calcified region and the subchondral bone. Tidemark is flexible and can move over time as a part of cartilage growth or remodeling in response to mechanical stress or injury and can therefore indicate tissue healthiness [6,7,8,9]. Vascular canals located in the subchondral bone region are narrow, ca. 10–40 µm in diameter, and a minor part of these are open to the calcified region. In 1990, Clark [10] showed that no vessels extended beyond the tidemark into the UC, confirming the tidemark’s role in blocking vascular invasion and preserving the integrity and functionality of the cartilage.

Current approaches for the treatment of OC tissue defects [11] involve surgical procedures, such as microfracturing, in which micro holes in the underlying subchondral bone are made to stimulate the inflow of stem cells and growth factors into the defect to stimulate the healing process, or mosaicplasty, a procedure for replacement damaged areas of bone−cartilage with healthy grafts taken from the patient in a mosaic pattern. However, the effectiveness of these methods is limited due to the formation of fibrocartilage that has impaired resilience and reduced mechanical characteristics compared to hyaline cartilage [12,13]. Alternative approaches involve the implantation of extracellular matrix (ECM)-based or cell-laden synthetic grafts for enhanced tissue regeneration and mechanical support [14]. Progress in 3D printing and bioprinting opened multiple opportunities for its integration into advanced fabrication methods and the development of biocompatible scaffolds and grafts for regenerative medicine purposes and tissue models for studying interactions at the osteochondral interface, disease modeling, and potential treatment assessments [15,16,17].

Naturally derived polymers are widely used in scaffold fabrication due to their high biocompatibility, adaptability, and ability to mimic the native ECM primarily composed of collagen [18]. Gelatin (Gel), a collagen derivative, has excellent biocompatibility and shear-thinning properties and is widely used in multiple applications in tissue engineering. However, Gel lacks sufficient mechanical strength and stability and is often combined with oxidized alginate (OxAlg)—a natural derivative with crosslinking functionality. In addition to covalent bonds created between aldehyde and lysine/hydroxylysine side chains, ionic interactions between positively charged amino acids and carboxyl groups of HAp and their contribution to the intermolecular interactions can sizably change rheological characteristics of the gel and the mechanical properties of the engineered construct [19,20]. Yet, these effects depend on the concentration of the dopant. Both chemical and physical crosslinking approaches within the matrix are promising for fabricating highly porous and biocompatible scaffolds [21]. Multiple studies on the development of osteochondral tissue models using 3D printing were published in the past few years, where mechanical and biological characteristics of the models were assessed, acknowledging the importance of inter-tissue interface characteristics [22,23,24,25]. The tidemark was addressed in some studies [26,27,28,29] yet was not evidenced, possibly because they have not specifically targeted tidemark regeneration in their efforts. In our comprehensive [30], we highlighted the importance of tidemark and its role in OC tissue, which is frequently overlooked despite its relevance.

Successful design of the scaffolds for OC regeneration studies and treatment purposes should aim to engineer materials with high similarity of mechanical characteristics and functionality of the tissue, such as support of cell migration and nutrient transport within the scaffold [31,32].

The development of bioresorbable polymeric grafts and porous scaffolds for regenerative medicine is challenging due to the complexity of native tissues [33]. To replicate these intricate structures, bone graft substitutes and scaffolds must have similar gradations. Functional grading of scaffolds, particularly when seeded with stem cells, helps mimic the transitions found at tissue interfaces like cartilage−bone and tendon−bone [34,35].

These technologies are scalable and can be produced under controlled conditions. Various extrusion-based methods, such as co-extrusion [36], hybrid extrusion [37], twin-screw extrusion with electrospinning [38,39], 3D printing [40], and melt electrowriting [41], have been successfully used to manufacture graded scaffolds.

Previous studies demonstrated development of the inks to fabricate highly interconnected scaffolds with controlled large and small pores also containing HAp to improve bioinductive and mechanical properties. [19,42,43] This design mimics the properties of natural cartilage, allowing the engineered scaffold to serve effectively as a model for cartilage tissue due to the strong resemblance between the scaffold’s characteristics and those of the natural tissue. The pores of the desired dimensions were previously formed using the cryopolymerization process [44], and a mesh to act as a tidemark [45] could be fabricated using electrospinning.

Accordingly, the clinical problem related to OA is that when remaining untreated, articular cartilage defects can progress to full thickness (or Grade IV) defects, which do not heal themselves due mainly to poor vascularization of the cartilage that is controlled by the selectively permeable tidemark. Current clinical approaches for the treatment of OC injuries are surgical interventions that disrupt the tidemark, allow blood vessel invasion together with mesenchymal stem cell migration, and lead to the formation of a fibrocartilage scar tissue (as a result of fibroblastic differentiation of mesenchymal stem cells in addition to other lineages), which degenerates easily and is compositionally different from and mechanically inferior to the articular cartilage. Regenerative engineering efforts have provided valuable outcomes however with poor focus on the tidemark, which is a critical component for a seamless function of the joint. Therefore, new approaches are needed for the design and fabrication of biomimetic scaffolds.

This study attempts for the first time to design and fabricate graded scaffolds to mimic the zones of AC, tidemark, CC, and SB at physiologically relevant dimensions (the cement line is not of special interest for this specific study). The findings will eventually serve as useful data for preclinical and translational studies aiming at OC repair/regeneration. The aims of this study are (i) characterizing the OC interface of rabbit knee, (ii) designing a continuously graded biomaterial scaffold with structural properties similar to native OC interface including the tidemark, and (iii) fabricating this scaffold using a hydrogel ink as material and 3D printing technology for processing. We hypothesize that the hydrogel graft fabricated using ink printing will mimic the structural and compositional properties of native OC interface.

The outcomes of this study are expected to have significant scientific, technological, and socio-economic contributions to the orthopedic research society. A significant outcome will be the existence of physiologically and structurally biomimetic grafts to be utilized in regenerating a fully functional OC interface. In a broader sense, the grafts to be fabricated in this study are likely to find clinical applications for more than 250 million people over the age of 50, worldwide.

## 2. Results and Discussion

### 2.1. Characterization of Inks

FTIR spectroscopy was utilized to verify the successful oxidation of sodium alginate and characterize the products. The spectra comparison highlighted a new peak at 1730 cm^−1^ in the oxidized product, which corresponds to the symmetrical vibration of the aldehyde group. Thus, the spectroscopy confirmed both the effective modification of alginate and the purity of the OxAlg compound (Figure S1).

In the Gel/OxAlg ink formulation, gelatin functions as the main structural component, offering shear-thinning properties required for optimal printing performance. OxAlg is crucial for enabling chemical crosslinking and enhancing ionic stability. The Gel to OxAlg ratio was determined from our earlier studies, aiming to balance uninterrupted extrusion during 3D printing with fiber uniformity and the desired mechanical and morphological characteristics of the final scaffold [20,46]. A 3.5% *w*/*w* concentration of HAp was used to substantially improve the mechanical properties of the scaffolds and promote osteogenesis while minimally affecting the ink’s viscoelasticity and printability [20].

**Figure 1:**
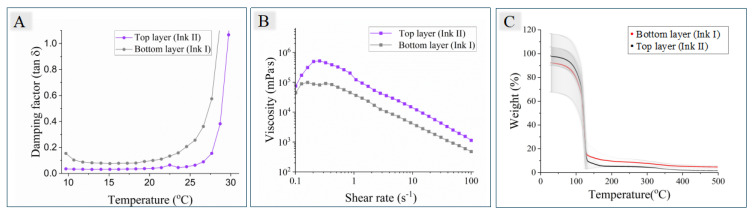
Rheological characterization of Inks I and II: (**A**) damping factor (tan δ) vs. temperature and (**B**) viscosity at variable shear rates at 15 °C. (**C**) TGA analysis for HAp content in inks.

The rheological characterization of formulated Inks I (OxAlg with HAp) and II (OxAlg) was performed to evaluate their suitability for extrusion-based 3D printing and refine the printing process. Figure 1A shows the dynamic mechanical analysis of the inks, with the damping factor (tan δ) derived from the ratio of loss modulus to storage modulus, illustrating the balance between viscous and elastic behavior across different temperatures. Both inks display the behavior characteristic of gelatin-based hydrogels, with predominantly elastic properties in the 15–30 °C range, followed by a noticeable increase at higher temperatures. Ink II shows a lower relative tan δ value than Ink I, suggesting a more pronounced gel-like behavior. Additionally, Ink II exhibits slightly greater elasticity at lower temperatures, with the storage modulus continuing to dominate below 15 °C.

The viscosity curves at varying shear rates at 15 °C in Figure 1B reveal that both inks have slopes close to −45°, indicating shear-thinning behavior, where the viscosity decreases as the shear rate increases. The flow curves show that incorporating HAp results in a slight decrease in viscosity across all shear rates. This reduction can be attributed to the well-dispersed HAp nanoparticles, which lower the overall resistance to flow while still allowing for smooth filament deposition comparable between Ink I and II. 

TGA was performed to confirm the presence of HAp in Ink I. Two types of inks were tested, one with HAp (Ink I) and one without (Ink II). Both inks showed an immediate weight reduction of around 100 °C, attributed to water evaporation (Figure 1C). At 500 °C, the remaining weight of the material without HAp decreased to 1.62 ± 0.06%. In contrast, the material containing HAp, representing the calcified region, retained 5.11 ± 0.08% of its weight. The difference of 3.49% between these values corresponds to the remaining non-decomposing components (HAp, β-glycerophosphate disodium salt hydrate, and polymers) in the HAp ink, which aligns with the initial formulation containing 3.5% *w*/*w* of HAp in Gel/OxAlg.

The rheometry data confirmed the suitability of the examined ink compositions for 3D printing, so they were used for layer-by-layer scaffold fabrication using an extrusion-based 3D printer.

### 2.2. Fabrication and Mechanical Characterization of OC Scaffolds 

**Scheme 2:**
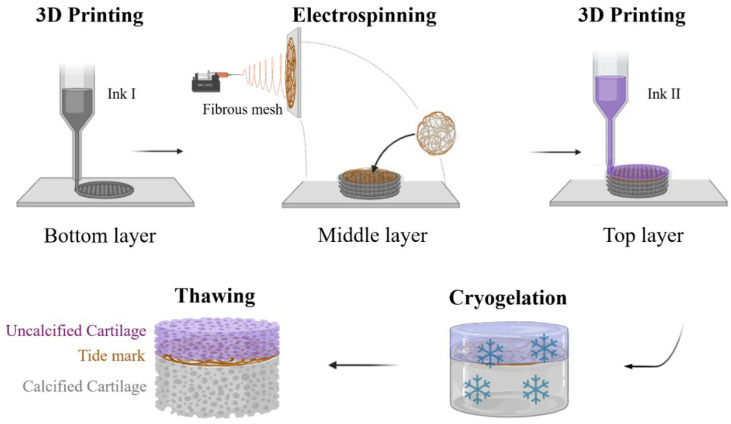
Steps in OC tissue model fabrication process, with tidemark membrane.

To fabricate the OC tissue model, namely, the region that contains a calcified and uncalcified interface with a tidemark barrier, Ink I, Gel/OxAlg/HAp, was deposited on a glass base in round shape geometry, using a 3D printer. The PCL-based fibrous mesh was fabricated via electrospinning, formatted into a circle with an 8 mm diameter, and transferred onto the 3D printed base immediately after the deposition. Next, Ink II, Gel/OxAlg was deposited onto the PCL membrane and moved to −20 °C for 24 h for cryogelation. After thawing, a 3-layer interconnected porous construct was revealed. Scheme 2 illustrates the steps in the scaffold’s fabrication. 

**Figure 2:**
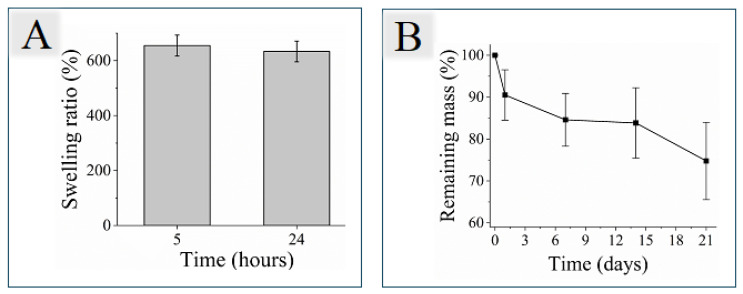
Characterization of the OS scaffolds. (**A**) The swelling capacity tested after 5 and 24 h. (**B**) The degradation rate of the scaffolds in PBS, at 37 °C. N = 3 samples of each type at each time point.

The scaffold’s physical attributes, including its ability to absorb fluids, are essential for controlling important factors in tissue growth, such as cell proliferation, nutrient and waste transport, degradation, and ECM production [47,48]. The results of the swelling test, shown in Figure 2A, indicate that the OS scaffold swelled by 654% after 5 h. By the end of the 24 h, the scaffold exhibited no significant additional swelling, stabilizing at 633%.

The scaffold’s degradation rate should align with the pace of new tissue formation, particularly during the critical first two to three weeks when cell proliferation, ECM secretion, and tissue formation are most active [49]. Maintaining mechanical stability during this period is vital for effective cell seeding and growth. This study conducted degradation testing by measuring weight loss over time, following a 24 h soak in PBS to ensure maximal swelling and better mimic in vivo conditions.

Figure 2B shows the results of the degradation test, where scaffold mass changes were tracked over 21 days. After 7 and 14 days, the OC scaffolds retained approximately 85% of its original mass, with around 75% remaining intact after 21 days. This gradual yet sustained degradation of the OS scaffolds reflects their resistance to hydrolysis and mechanical robustness, which is consistent with our previous findings [20,46].

### 2.3. Morphological Characterization of OC Scaffolds

**Figure 3:**
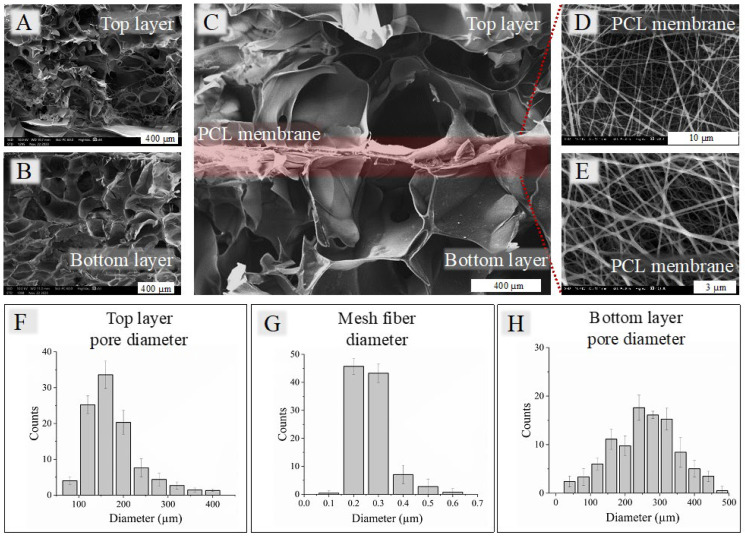
SEM micrographs corresponded to: (**A**) top layer fabricated from Gel/OxAlg 4/6% *w*/*w*, (**B**) bottom layer fabricated from Gel/OxAlg/HAp 4.8/5/3.5% *w*/*w*, (**C**) magnified image of entire scaffold, (**D**) and (**E**) electrospun PCl membrane (mesh) at different magnifications. Pore size distribution histograms of top hydrogel layer (**F**), fiber diameter distribution of PCL membrane (**G**), and pore size distribution histograms of bottom layer (**H**).

SEM cross-sectional views of the OC fabricated scaffold are presented in Figure 3A–E. Figure 3C distinguishes the three fused layers with a noticeable PCL membrane interface representing the tidemark. The cross-sectional views of the top and bottom layers (Figure 3A,B) demonstrate interconnected porous morphology with different pore diameters, while the fibrous structure of the electrospun mesh can be seen in Figure 3D,E.

Histograms in Figure 3F–H show the respective pore size distribution within the scaffold layers, with an average of 170 µm and 250 µm for the top and bottom layers, respectively. Figure 3F illustrates the pore diameter profiles for the top layer produced from Ink II, revealing a monomodal, right-skewed Gaussian distribution with pore sizes ranging from 50 µm to 400 µm. In contrast, the bottom layer, made from Ink I, displayed a more homogenous distribution with a broader range of pore sizes, from 40 µm to 500 µm (Figure 3H). These pore size and distribution differences can be attributed to the varying amounts of hydrogel precursor materials in the two layers. The higher Gel content in Ink II increases viscosity, which hinders diffusion, leading to smaller average pore sizes and higher pore density. Conversely, the lower viscosity and higher concentration of the crosslinking agent in Ink I may contribute to a broader range of pore sizes. Additionally, the presence of HAp nanoparticles can affect the gel structure by absorbing water, reducing the efficiency of polymer-water phase separation during cryogelation, and further contributing to the variation in pore sizes.

Matrix morphology, especially its porosity, is an essential characteristic of hydrogel scaffolds that significantly impacts the efficiency and speed of various biological processes. Hydrogel matrices with pore sizes larger than 100 μm promote enhanced cartilage, bone regeneration, and capillary growth by allowing unrestricted cell infiltration and proliferation [50]. Additionally, larger pores enhance the supply of oxygen and nutrients, leading to improved stem cell differentiation [51].

The electrospun PCL mesh, designed to mimic the tidemark, had fiber diameters and pore sizes ranging from 0.1 µm to 0.7 µm (Figure 3G). In native tissue, the tidemark is a 5–10 µm-thick semipermeable boundary that not only separates CC from UC but also serves as a barrier to prevent blood vessels from invading the uncalcified region. The small pore size of the electrospun mesh allows it to effectively replicate the function of the tidemark as a selective membrane.

### 2.4. Structural and Mechanical Characterization of Native OC Tissue vs. Fabricated OC Scaffold

**Figure 4:**
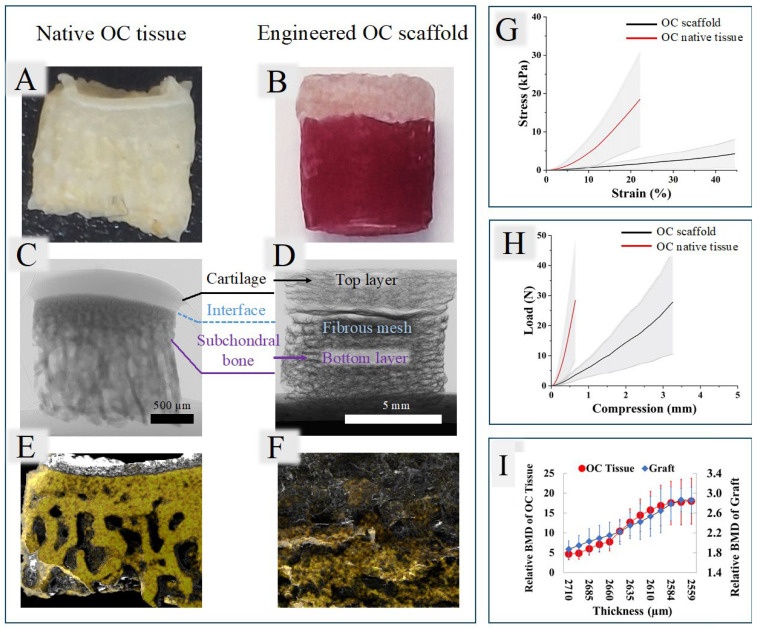
Mineral presence and distribution in native OC tissue and scaffolds. Optical images of (**A**) native OC tissue and (**B**) engineered OC scaffolds. Reconstructed micro-CT images of (**C**) native OC tissue and (**D**) engineered OC scaffolds. EDX elemental mapping of (**E**) native OC tissue and (**F**) engineered OC scaffolds. (**G**) Stress–strain curve, (**H**) load compression graph of native OC tissue and scaffolds, and (**I**) the distance of gradient in the grafts compared to the native OC interface. Shaded regions represent error bars (error bars = standard deviation).

Native OC tissue from the knee joints of rabbits was used to validate the fabricated OC scaffold model (Figure 4A,B). Reconstructed micro-CT images of both the native OC tissue (Figure 4C) and the engineered OC scaffold (Figure 4D) reveal structural similarities, with each displaying a cartilage region on top, an interface, and a calcified region at the bottom. The layers of cartilage and calcified regions can be visually and qualitatively distinguished in both the native OC tissue and the scaffold. BMD was calculated for both samples within the interfacial region (Figure 4I), showing a similar trend of increasing BMD from the cartilage region at the top to the calcified region at the bottom. The mineral density plateaued at both ends, with a gradual transition observed within the interface region. The gradient thickness of mineral concentration was found to be 151 µm for grafts and the native OC interface, with the native OC tissue exhibiting higher mineral density (ranging from 4.44 ± 1.44 to 17.98 ± 5.85) compared to the scaffold (ranging from 1.80 ± 0.17 to 2.86 ± 0.16).

Additionally, mineral presence and distribution were assessed using EDX mapping. Elemental mapping for phosphorus (P) and calcium (Ca), depicted in orange and yellow, respectively, showed mineral presence in the calcified region of the native tissue. In contrast, no minerals were detected in the cartilage region (Figure 4E). Similarly, EDX mapping of the fabricated OC scaffold (Figure 4F) confirmed the presence of minerals in the bottom layer, corresponding to the calcified region, and the absence of minerals in the top layer, corresponding to the cartilage region.

Compression tests were conducted on the native OC tissues and fabricated scaffold (Figure 4G,H). The stress–strain response of the native OC tissue showed it could withstand a compressive stress of 24.4 ± 3.9 MPa when strained to 14.4 ± 0.03%, while the scaffold endured a stress of 2.7 ± 1.8 MPa (Table S1). Notably, the scaffold exhibited a greater strain capacity, reaching an ultimate value of 37.3 ± 3.8%. The moduli of the native OC tissue and the scaffold were measured at 1.8 ± 0.1 MPa and 0.07 ± 0.05 MPa, respectively. Regarding load-compression characteristics, a load of 37.4 ± 6.3 N was required to compress the OC tissue by 0.6 mm, whereas the scaffold required a load of 21.0 ± 9.0 N to achieve complete deformation at 2.6 mm compression. The OC tissue and scaffold stiffness values were 83.3 ± 1.9 N/mm and 5.60 ± 2.85 N/mm, respectively.

### 2.5. Cell Viability Data and Proliferation on Fabricated OC Scaffolds

An electrospun PCL membrane in OC scaffold was integrated to mimic the barrier at the interface of subchondral bone and cartilage, blocking vascular and cellular invasion to preserve the integrity of the cartilage [52,53].

**Figure 5:**
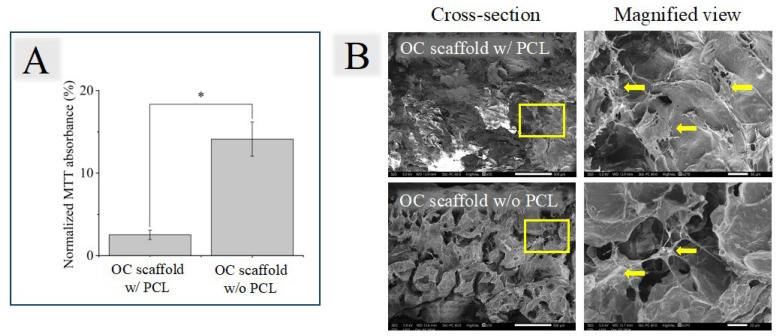
(**A**) The spectral absorbance of MTT from cells remaining in the well after 24 h of culturing. The values were normalized against the control cells seeded on the bottom of the well. Each group had the same initial cell population of 2 × 10^5^ cells per well. An asterisk (*) indicates a significant difference with *p* ≤ 0.05. (**B**) The SEM images of scaffolds containing cells after 14 days of culturing, along with the corresponding high-magnification images from the yellow box. The yellow arrows highlight the cells. The scale bars represent 500 µm and 50 µm for the magnified view.

To estimate the migration of cells through PCL mesh, OC scaffolds with and without PCL mesh were placed in a 12-well plate, seeded with the same amount of rMSCs, and incubated in the same conditions as the control group, where cells were seeded at the bottom of the wells. After 24 h, the scaffolds were removed from the wells, and the metabolic activity of the cells that remained in the respective wells was measured. Values were normalized to the control wells (97.52% ± 0.48), revealing an average of 2.52% and 14.12% for the OC scaffolds with PCL and scaffolds without PCL, respectively (Figure 5A). The reduced metabolic activity response after the removal of the OC scaffolds was attributed to the partial blockade of cells by the mesh and adhesion to the scaffold, revealing the effectiveness of PCL mesh in OC scaffold to partially block cells and mimic tidemark. 

Figure 5B presents a cross-sectional view of the upper portion of the OC scaffold and the scaffold without PCL, with the magnified regions indicated by yellow boxes. In the upper layers of the OC scaffold, individual well-spread cells are visible, forming a distinct layer, as marked by yellow arrows. In contrast, the scaffold without PCL shows only a few cells in the top layer. This observation is consistent with the findings presented in Figure 5A.

**Figure 6:**
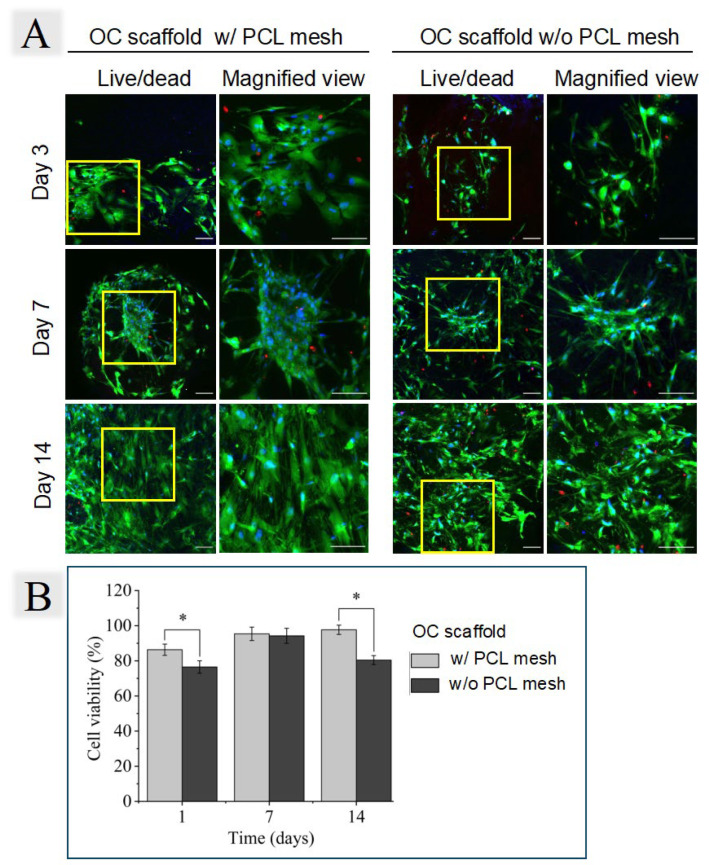
In vitro scaffold characterization was conducted, with representative images captured through Z-stack scanning during the live/dead staining assay. These images depict (**A**) the OC scaffold with and without PCL mesh on days 1, 7, and 14 post-cell seeding. Live cells are shown in green, nuclei in blue, and dead cells in red. Zen lite software (ZEN 3.6 blue edition) was used to enhance the colors for improved visualization. The scale bar indicates 100 µm. (**B**) The quantitative analysis of cell viability in the OC scaffolds after 1, 7, and 14 days. An asterisk (*) indicates a significant difference with *p* ≤ 0.05. (w/: with, w/o: without). Yellow box in the Live/dead column is magnified as shown in the Magnified view.

To confirm that the hydrogel material did not exhibit cytotoxicity or negatively affect cell viability, live/dead staining was performed on the fabricated scaffolds. Figure 6A displays images of live/dead staining on days 3, 7, and 14. In these images, the green channel represents live cells, the red channel shows dead cells, and the blue channel highlights cell nuclei. The merged images provide a comprehensive view of all three channels, allowing for detailed visualization of the cellular morphology within the OC scaffolds.

During the experiments, distinct cell morphologies were observed within the scaffolds. On day 3, cells in both scaffolds exhibited a spherical shape and formed clusters. However, cell distribution was notably more uniform on the OC scaffold compared to the scaffold without PCL. By day 7, significant cell proliferation and spreading were evident on both scaffold types, though differences in cell shape were apparent, with a significantly higher number of cells on the OC scaffold with PCL, while cell growth was more limited on the scaffold without PCL. By day 14, a dense population of live cells was observed for the OC scaffold with PCL. This suggests that the presence of the PCL mesh may contribute to trapping more cells in the upper part of the OC scaffold.

Figure 6B summarizes the quantitative results of a two-week viability test conducted on stem cells seeded onto the OC scaffolds with PCL mesh and scaffolds without PCL, with images captured using a confocal laser-scanning microscope. Results indicate high cell viability of approximately 85% or higher for the OC scaffold with PCL mesh and around 75% for the OC scaffold without PCL mesh on days 3 and 14. On day 7, scaffolds without PCL showed similar viability to those without the mesh. Although the OC scaffold with PCL had a higher cell density than the scaffold without PCL, this difference can be attributed to the slightly higher number of cells captured by the PCL mesh. These findings are in agreement with what is recommended for a candidate biomaterial [54].

## 3. Conclusions

A significant challenge in designing multi-layered/graded constructs is achieving the right mechanical properties and biological functionality across the different layers, ensuring not only compatibility with the host tissue but also effective integration and function over time. The OC graft fabricated using combined printing and electrospinning techniques demonstrated the potential to serve as a biomimetic hydrogel filler for regenerating OC defects to restore the function of the knee joint. A physiologically relevant thickness gradient of mineral concentration has been achieved in a controlled manner. The PCL layer effectively mimicked the tidemark, a key structural element in OC tissue, preventing cellular migration and ensuring tissue separation. It is expected that the proposed osteochondral graft will be effectively used to address a significant clinical problem that affects millions of people worldwide, with significant societal and economic impacts.

## 4. Materials and Methods

### 4.1. Materials

The following materials were used in this study: hydroxyapatite (677418-10G Sigma-Aldrich, St. Louis, MI, USA, MKBS2145V), polycaprolactone MW = 80,000 g/mol (440744 Sigma-Aldrich, St. Louis, Missouri, United States, MKCL0177), acetic acid (695092 Sigma-Aldrich, St. Louis, MI, USA, STBH0410), pyridine (270970 Sigma-Aldrich, St. Louis, MO, USA, STBJ3354), formic acid (1.10854 Sigma-Aldrich, St. Louis, Missouri, United States, Z0550154 908), glutaraldehyde (Nr-4995.1 Roth, UN2927), β-glycerophosphate disodium salt hydrate (G9422-50G Sigma-Aldrich, St. Louis, MI, USA, SLCF2153), glycine (1610718 Bio-Rad, Hercules, CA, USA, 64331789), ethylene glycol (324558-1L Sigma-Aldrich, St. Louis, Missouri, United States, SHBK8330), phosphate-buffer saline (P4417 Sigma-Aldrich, St. Louis, MI, USA, SLBW7839), formalin (HT501128-4L Sigma-Aldrich, St. Louis, MI, USA, MKCG3336), sodium periodate (S-1878 Sigma-Aldrich, St. Louis, MI, USA, 23J094110), alginic acid sodium salt from brown algae (#A2033 Sigma-Aldrich, St. Louis, Missouri, United States, SLCD1535), gelatin from porcine skin (gel strength 300, Type A, #G2500 Sigma-Aldrich, St. Louis, MI, USA, SLCG4328), and sodium borohydride (#S0480 Tokyo Chemical Industry, Tokyo, Japan, YDTOG-PY). Calcein AM (ab141420, APN20034-1-2), ethidium homodimer (ab145323, GR3192625-47), and Hoechst 33342 (ab228551, GR3436460-2) were purchased from Abcam, Cambridge, UK. Dulbecco’s modified eagle’s medium, high glucose (DMEM, #D6429, RNBG7302), Dulbecco’s modified eagle’s medium, high glucose without phenol red (#D1145 Sigma-Aldrich, St. Louis, MI, USA, RNBL6320), non-essential amino acid solution 100× (NEAA, #M7145, RNBJ2359), L-ascorbic acid 2-phosphate sesquimagnesium salt hydrate 95% (#A8960, SLCL1084), trypan blue solution (#T8154, RNBK6727), and dimethyl sulfoxide (DMSO, #472301, MKCH9220) were purchased from Sigma-Aldrich, St. Louis, MI, USA. Fetal bovine serum (FBS, #FBS-HI-22A, CP21-4529-HI) was purchased from Capricorn Scientific (Ebsdorfergrund, Germany), 0.5% trypsin-EDTA solution 10× (#sc-363354, MS01EF) was purchased from ChemCruz (Huissen, The Netherlands), dexamethasone water soluble (BML-EI126-0001, 09131935) was purchased from Enzo (Farmingdale, NY, USA), and MTT labeling reagent (#11465007001, 11693506) was purchased from Roche (Basel, Switzerland).

Mesenchymal stem cells from rats (rMSCs), obtained from the bone marrow of healthy donor animals, were provided by the National Center of Biotechnology in Kazakhstan. The isolation of rMSCs was approved by the Local Ethics Committee as well as the Institutional Review Board of the National Center for Biotechnology (IRB 00013497).

### 4.2. Ink Formulations

Oxidized alginate (OxAlg) was synthesized using a standard protocol [46]. In particular, 1.8 g of sodium alginate was dissolved in 165 mL of DI water (bioSan, Model: LabAqua, Riga, Latvia) at room temperature. The parameters of the purifier were set to 0.1 μS/cm conductivity, <5 ppb total organic carbon, and <1/mL particles above 0.22 µm. Next, 0.18 g of sodium periodate was added and stirred in a light-proof chamber for 1 h to oxidize the alginate. The reaction was terminated by adding 0.25 mL of ethylene glycol and stirring for 30 min. The solution was dialyzed against DI water for 24 h with a 15 kDa molecular weight cut-off dialysis membrane. To confirm successful oxidation, the obtained product was analyzed using Fourier transform infrared (FTIR) spectroscopy (Nicolet iS10, Thermo Scientific, Waltham, Massachusetts, United States). OxAlg was lyophilized. The product was concentrated to a final concentration of 8% (*weight*/*volume*) and stored at 4 °C. A gelatin component was prepared at 12% (*weight*/*volume*) and stored at 4 °C.

For the preparation of Ink I, Gel/OxAlg/HAp, Gel, and OxAlg precursors were mixed to obtain 4/6% (*weight*/*weight*), and 3.5% HAp (*weight*/*weight*) was added. Ink II, Gel/OxAlg, was prepared by mixing Gel and OxAlg at final concentrations of 4.8/5% (*weight*/*weight*), respectively. To enhance cell viability, both inks contained 0.3 M disodium β-glycerophosphate. All inks were vortexed for 30 s and homogenized in a water bath at 45 °C for 3 min. The Gel to OxAlg ratio was determined as per our prior experiments [19], aiming at a balance between uninterrupted extrusion during 3D printing and homogeneity of fibers and the desired mechanical and morphological properties of the resulting scaffold.

### 4.3. Rheological and Thermogravimetric Analysis 

An oscillatory test was performed using Anton Paar, MCR302 (Tokyo, Japan) rheometer to assess the temperature dependence of the storage modulus (G′) and loss modulus (G′′) of the inks across temperatures ranging from 10 to 40 °C (n = 3/group). The experiment was performed at a heating rate of 1 °C/min, a frequency of 1 Hz, and a shear strain of 1%. The shear-dependent viscosities were also evaluated using a strain sweep test from 0.1 to 100 1/s (n = 3/group). Thermogravimetric analysis (Netzsch DSC-TGA, Selb, Germany) was performed on both Inks I and II by heating them from room temperature to 500 °C at 25 °C/min (n = 3/group).

### 4.4. Scaffold Fabrication

#### 4.4.1. PCL Mesh Layer

Tidemark-mimicking nanofiber mesh was fabricated via electrospinning using 0.8 g of PCL dissolved in 1 mL of a 1:1 (*volume*/*volume*) mixture of formic acid and acetic acid, along with 0.006 mL of pyridine. The precursors were stirred at 40 °C for 2 h and loaded into a 5 mL syringe equipped with a 21 G blunt needle. The electrospinning process was carried out at 9 kV for 10 h with distance d = 7 cm between the needle tip and the collecting plate. The fibrous mesh was allowed to dry at room temperature for 24 h, and its geometry was adjusted to fit the circle (d = 8 mm).

#### 4.4.2. Hydrogel Layers and Incorporation of PCL Mesh

Two ink formulations were loaded into separate syringes, cooled for 1 min in an ice bath, and installed into two separate temperature-controlled print heads of the BioX 3D printer CELLINK (Göteborg, Sweden), kept at 15 °C. In contrast, the print bed temperature was set to 4 °C. Attached to the syringes were 27 G needles. To fabricate the bottom layer of the scaffold, 5 layers of Ink I, Gel/OxAlg/HAp, were deposited in a checkmate pattern with diameter d = 8 mm on a glass substrate, using an extrusion pressure of 80 kPa and printing speed of 5 mm/s. The settings were optimized to achieve a uniform extrusion of 0.45 mm-thick fibers. After the deposition of the bottom layer, an electrospun PCL membrane of 8 mm in diameter was placed onto the freshly printed base layer and covered by 5 layers of Ink II using the same printing parameters.

After 3D printing, the fabricated scaffold was placed at −20 °C for 24 h for cryogelation, followed by treatment with a 1% glutaraldehyde solution for 2 h to enhance its mechanical proper. The construct was treated with 2% glycine and 50 mM sodium borohydride to neutralize residual aldehyde groups and Schiff bases. All scaffolds were thoroughly washed with PBS, lyophilized, and stored at 4 °C until further use.

### 4.5. Scaffold Characterization

#### 4.5.1. Morphology by SEM

After lyophilization, the scaffold morphology was analyzed via scanning electron microscopy (SEM) on a JSM-IT200 (Jeol, Tokyo, Japan) at accelerating voltages of 10 kV and 5 kV for PCL mesh and printed region, respectively, with a probe current of 40 pA and a 7 nm gold coating. As previously described, pore and fiber diameters were measured using ImageJ Version 1.54g (NIH, Bethesda, Maryland, United States). In particular, ten evenly spaced lines were superimposed on SEM images, and the diameters of fibers and pores intersecting these lines were recorded to obtain measurements.

#### 4.5.2. Swelling and Degradation Tests

The freeze-dried scaffolds were weighed to determine their dry mass *W*(*o*) before being immersed in a 10 mM PBS solution for 24 h. At designated intervals (5 and 24 h), the swollen scaffolds were removed and reweighed to obtain their final mass *W*(*f*) (n = 3). The swelling capacity was then calculated using the following equation:

(1) $$\mathrm{Swelling}\;\mathrm{capacity}\;(\%)=\;\frac{W(f)-W\left(o\right)}{W(f)}\;\times 100$$

Each scaffold’s initial dry mass *W*(*o*) was recorded for degradation analysis. The samples were then immersed in PBS and incubated at 37 °C with 5% CO₂ for 21 days. At intervals (1, 7, 14, and 21 days), the samples were washed with DI water to remove residual PBS, lyophilized overnight, and reweighed to determine the final mass *W*(*f*). The degradation rate was calculated using the following equation (n = 3):

(2) $$\mathrm{Degradation}\;\mathrm{rate}\;(\%)=\frac{W(o)-W\left(f\right)}{W(o)}\times 100$$

### 4.6. Characterization of Native OC Tissue

#### 4.6.1. Native OC Tissue Harvesting

The rabbit joints (8–10-month-old male) were obtained from a local abattoir, and the OC tissues were harvested in the form of cylindrical plugs (from the femoral bone) with dimensions of 3 mm × 3 mm (diameter × height).

#### 4.6.2. Mechanical Testing by Compression

The mechanical properties of the OC tissue and fabricated scaffolds were evaluated using a texture analyzer (TA.XTplusC, Stable Micro Systems, Surrey, UK) equipped with a 50 mm diameter plunger and a 0.5 kg load cell. The plunger applied compression at a 3 mm/min crosshead speed until complete deformation (n = 5/group). The resulting stress–strain and load-compression curves were graphically plotted based on the averaged values.

#### 4.6.3. OC Native Tissue Morphology and EDX Analysis

The morphology of the native OC tissue was examined using SEM (JSM-IT200, JEOL, Tokyo, Japan). To prepare the OC tissue for imaging, samples collected from rabbit knee joints were placed in saline solution for 2 h at 4 °C, then fixed in 10% formalin overnight. The samples were dehydrated through a graded ethanol series, dried, and coated with 7 nm gold before SEM. 

Additionally, Energy Dispersive X-ray (EDX) evaluation was performed on the fabricated scaffolds and native tissues to assess mineral content and distribution. The analysis was performed with an accelerating voltage of 20 kV and a probe current of 60 pA.

#### 4.6.4. Micro-CT Analysis 

Freshly harvested OC tissue plugs were immediately subjected to testing. Scanning can take several hours depending on the desired resolution, so the specimens were maintained in a hydrated state using a custom-designed chamber throughout the process. Scans were conducted using a SkyScan 1272 micro-CT system (Bruker, Billerica, MA, USA) at 60 kV, 60 µA, with 2 × 2 binning, an Al filter of 0.5 mm, and a total scan time of 16 h. Three-dimensional reconstruction and bone mineral density (BMD) analysis were performed using Bruker’s CTVox (v3.2) and CTAn software (Ver 1.18.8.0).

### 4.7. Cell Culture

At passage two, rat mesenchymal stem cells rMSCs were harvested and cultured in an expansion medium containing DMEM, 10% fetal bovine serum (FBS), 1% penicillin/streptomycin/fungizone, 1% non-essential amino acids (NEAA), and 0.001% basic fibroblast growth factor (bFGF), with the medium being replaced every three days. Cell detachment for passaging was achieved using 0.5% trypsin-EDTA. To induce osteogenic differentiation, rMSCs were exposed to an osteogenic medium. This medium was based on a complete medium of DMEM, 10% FBS, and 1% penicillin/streptomycin/fungizone. For osteogenic induction, β-glycerophosphate disodium salt hydrate (10 mM), dexamethasone (100 nM) and l-ascorbic acid-2-phosphate sesquimagnesium salt hydrate (50 μg/mL) were added to the complete medium. The resulting medium was filtered using a 0.22 μm filter and applied to the scaffolds following rMSC seeding. The osteogenic medium was refreshed every three days for three weeks.

#### 4.7.1. Cell Viability and Morphology

To assess cell viability and morphology, cells were seeded at a density of 2 × 10^5^ cells/scaffold and cultured at 37 °C, 5% CO_2_. The environment was humidified using a water bath in the incubator. Before seeding, the scaffolds were ethanol-sterilized, rinsed with PBS, and immersed in a complete medium. The scaffolds were then placed in an incubator for 2 h to allow for cell adhesion, after which osteogenic media was added. On days 1, 7, and 14, the scaffolds were rinsed with PBS and stained with complete medium and fluorescent dyes: Hoechst 33342 for nuclei staining, Calcein AM for live cells, and Ethidium homodimer for dead cells. After staining, the scaffolds were incubated for 40 min at 37 °C, washed with PBS, and transferred to imaging chambers (ibidi, μ-Slide) containing a complete medium. Imaging was performed using an 10× objective with excitation channels at 350/461 nm for Hoechst, 404/517 nm for Calcein, and 517/617 nm for Ethidium homodimer on a confocal laser-scanning microscope (LSM 780, ZEISS, Oberkochen, Germany). Live and dead cells, labeled as #Live and #Dead, respectively, were counted using ImageJ (Fiji) software (Version 1.52p), and cell viability was calculated using the following formula:

(3) $$\mathrm{Cell}\;\mathrm{viability}\;(\%)=\frac{\#\mathrm{Live}}{\#\mathrm{Live}+\#\mathrm{Dead}}\times 100\%$$

#### 4.7.2. Monitoring Cell Migration Through Hydrogel Layers and PCL Mesh

Cells were seeded onto scaffolds with and without PCL mesh at a density of 2 × 10^5^ cells per scaffold. After 24 h, the scaffolds were transferred to a new well plate, and an MTT assay (3-(4, 5-dimethyl thiazol-2-yl)-2, 5-diphenyltetrazolium bromide) was performed to determine the unattached cells remaining in the wells. Control wells with the same initial number of cultured cells were used as references. A mixture of media and MTT solution in a 10:1 ratio was added to the empty wells and incubated for 4 h at 37 °C. Following incubation, 75% of the medium MTT mixture was carefully removed and replaced with DMSO to dissolve the formazan crystals. Absorbance was measured at 570 nm using a microplate reader (Varioskan Flash, Thermo Fisher Scientific, Waltham, MA, United States).

#### 4.7.3. SEM Imaging of Scaffolds with Cells

On days 1, 7, and 14 of culture, the cell-laden scaffolds were transferred from the medium, rinsed with PBS, and fixed with a 10% formalin solution for 10 min at room temperature. After fixation, the samples were washed with PBS, rinsed with distilled water, frozen in liquid nitrogen, and lyophilized overnight. The scaffolds were coated with a 7 nm gold layer for SEM imaging using an automatic sputter coater (turbomolecular pumped coater Q150T, Quorum Technologies, Lewes, UK). The presence and morphology of cells were analyzed using a scanning electron microscope (SEM, JSM-IT200, JEOL, Tokyo, Japan). Micrographs were captured at an accelerating voltage of 5 kV, a working distance of 12 mm, and a probe current of 30 pA.

### 4.8. Statistical Analysis 

The quantitative data are presented as mean ± standard deviation. Statistical comparisons were performed using ANOVA and Tukey’s post hoc test for pairwise analysis. Significance was defined at *p* < 0.05. All analyses were conducted using Origin software (Version 9.9.0.225). 

## Data Availability

All the data collected during the study are included in this manuscript.

## Supplementary Materials

The following supporting information can be downloaded at https://www.mdpi.com/article/10.3390/gels10110745/s1: Figure S1: The FTIR spectra for oxidized and untreated alginate are displayed; Figure S2: Photos of OC scaffolds fabricated using sequential printing; Table S1: Mechanical characterization of OC scaffold.
