# Supplementary material for: In Vitro Investigation of 3D Printed Hydrogel Scaffolds with Electrospun Tidemark Component for Modeling Osteochondral Interface
Source: Gels. 2024 Nov 15;10(11):745. doi: 10.3390/gels10110745 (PMC11593412; doi:10.3390/gels10110745)
Supplement: Supplementary file 1 [file gels-10-00745-s001.zip › gels-3283400-supplementary.pdf]

3D Printed Hydrogel Scaffolds with Electrospun Tidemark Component for Modeling the Osteochondral Interface

Supporting Information

Victoria Effiong Effanga[1], Dana Akilbekova[1], Fariza Mukasheva[1], Xiao Zhao[2], Dilhan M. Kalyon[2], and Cevat Erisken*[1]

[1] Department of Chemical and Materials Engineering, School of Engineering and Digital Sciences, Nazarbayev University, 010000 Astana, Kazakhstan.

[2] Department of Chemical Engineering and Material Science, Stevens Institute of Technology, NJ 07030, Hoboken, USA.

*Corresponding author, email: cevat.erisken@nu.edu.kz

Keywords: 3D printing, electrospinning, cryogelation, osteochondral interface, tissue engineering.

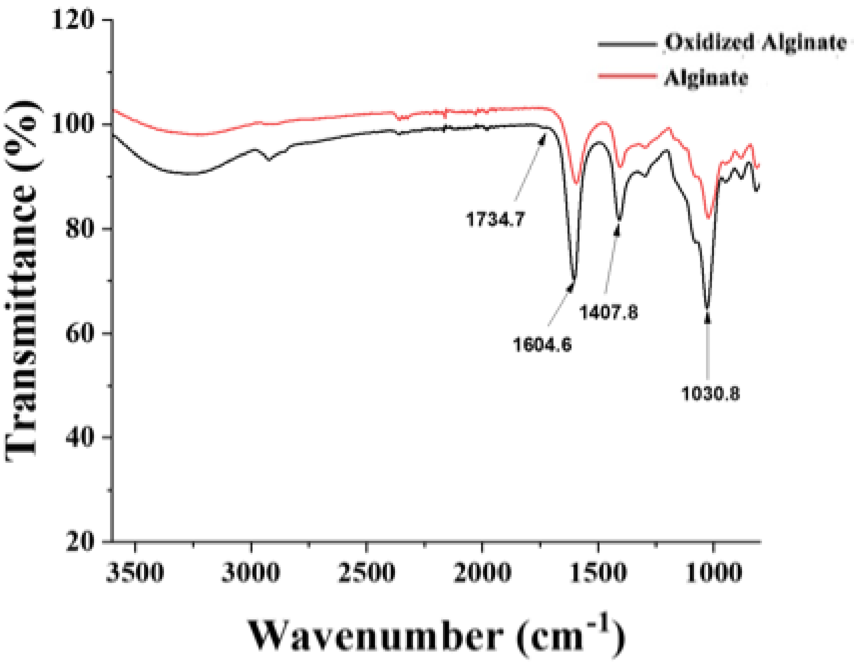

Figure S1: The FTIR spectra for oxidized and untreated alginate are displayed. Arrow at 1734.7 highlights the regions associated with the C=O bond stretching in the aldehyde group, indicating the successful production of oxidized alginate.

Table 1S. Mechanical characterization of OC scaffold.

	Stress at Break (MPa)	Strain at Break (%)	Modulus (MPa)	Stiffness (N/mm)	Load at Break (N)	Strain at Break (mm)
**OC Tissue**	24.4±3.9*	14.4±0.03*	1.8±0.1*	83.3±1.9*	37.4±6.3*	0.6±0.0*
**Graft**	2.7±1.8	37.3±3.8	0.07±0.05	5.60±2.85	21.0 ±9.0	2.6 ±0.0

* Difference at p<0.05

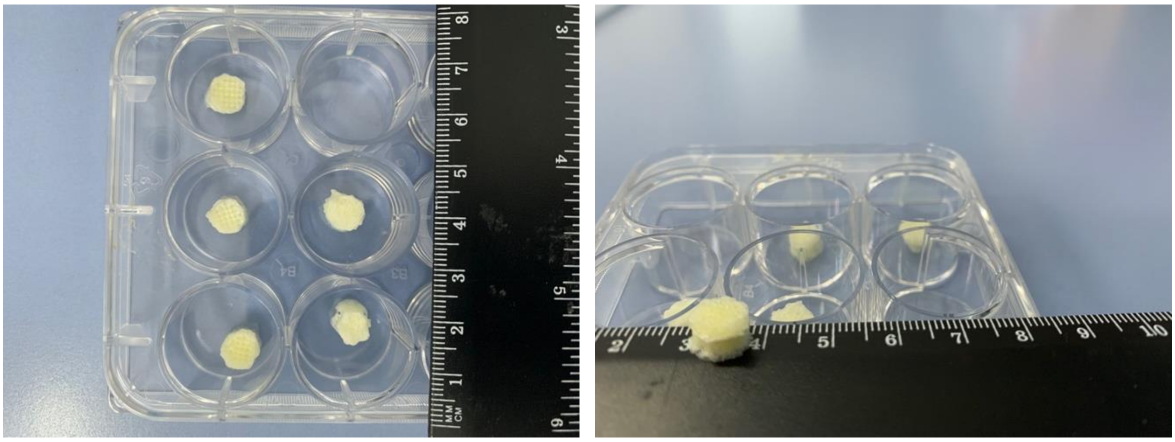

Figure S2: Photos of OC scaffolds fabricated using sequential printing.
